# 

**Published:** 1881-09

**Authors:** 


### Table of Contents,

### REPORTS OF SOCIETIES.

American Neurological Association.......... 321
The Centennial Anniversary of the Massachusetts Medical Society... 329
Causes of Death in Acute Pneumonia.......... 335

### SELECTIONS.

Prevention of Heat-Stroke.......... 341
Criminal Lunatics.......... 345
Alcohol: Its Therapeutical Uses, Internally and Externally.......... 347
Sensibility—Neuroses of the Pharynx and Larynx.......... 357
Specialism in Medicine.......... 365
Is Alcohol a Food?—When Should Malt Liquors be Preferred to Wines and Spirits in the Treatment of Disease? etc., etc.......... 366
Discussion upon Alcohol in the Philadelphia County Medical Society 370

### EXCERPTA.

On Disease of the Prostate.......... 376
Treatment of Ozæna.......... 377
Treatment of Incontinence of Urine in Women.......... 379
Sea-Sickness.......... 381

The Journal—New Arrangement.......... 383

